# *Dendropanax morbifera* Protects against Renal Fibrosis in Streptozotocin-Induced Diabetic Rats

**DOI:** 10.3390/antiox9010084

**Published:** 2020-01-19

**Authors:** Richa Sachan, Amit Kundu, Prasanta Dey, Ji Yeon Son, Kyeong Seok Kim, Da Eun Lee, Hae Ri Kim, Jae Hyeon Park, Su Hyun Lee, Jung-Hwan Kim, Shugeng Cao, Byung Mu Lee, Jong Hwan Kwak, Hyung Sik Kim

**Affiliations:** 1 School of Pharmacy, Sungkyunkwan University, 2066, Seobu-ro, Jangan-gu, Suwon 16419, Korea; richa.psit2009@gmail.com (R.S.); amitjupcl@gmail.com (A.K.); deyprasantadey@yahoo.com (P.D.); twiase@naver.com (J.Y.S.); kyeongseok@skku.edu (K.S.K.); bangu94@skku.edu (D.E.L.); kimhaeri56@daum.net (H.R.K.); sky3640@naver.com (J.H.P.); lydia7334@skku.edu (S.H.L.); bmlee@skku.edu (B.M.L.); 2 Department of Pharmacology, College of Medicine, Institute of Health Sciences, Gyeongsang National University, Jinju 52727, Korea; junghwan.kim@gnu.ac.kr; 3 Department of Pharmaceutical Sciences, Daniel K. Inouye College of Pharmacy, University of Hawai’i at Hilo, 200 West Kawili Street, Hilo, HI 96720, USA; scao@hawaii.edu

**Keywords:** *Dendropanax morbifera*, streptozotocin, diabetic nephropathy, inflammation, oxidative stress

## Abstract

The aquatic extract of *Dendropanax morbifera* (DP) is typically consumed as a beverage in Korea and China and is also used in various traditional medicines. However, the functional role of DP on diabetes-induced renal fibrosis is unclear. Here, the protective effects of DP extract against diabetes-induced renal fibrosis were evaluated. Streptozotocin (STZ, 60 mg/kg) was injected intraperitoneally in rats to induce diabetes. After 5 days, DP extract (25 mg/kg/day) and metformin (50 mg/kg/day) were administered orally to diabetic rats for 28 days. DP administration protected both body and organ weight loss in STZ-treated diabetic rats. Significant improvements in serum blood urea nitrogen (BUN), creatinine, and oxidative stress parameters were observed in diabetic rats by DP administration. DP extract markedly protected diabetic-induced histopathological damages in the kidney and pancreas. A significant reduction was observed in microalbumin, kidney injury molecule-1 (KIM-1), selenium binding protein-1 (SBP1), and pyruvate kinase muscle isozyme M2 (PKM2) levels in the urinary excretion of diabetic rats after the administration of DP extract. The expression of pro-inflammatory cytokines and fibrosis marker levels were significantly reduced in the kidney of diabetic rats. Our results strongly indicate that DP extract exhibits protective activity against diabetes-induced renal fibrosis through ameliorating oxidative stress and inflammation. Therefore, we suggest that DP extract can be used as a preventive agent on the progression of diabetic nephropathy and renal fibrosis.

## 1. Introduction

Diabetes is a metabolic disorder that occurs because of insufficient secretion or the absence of insulin in the body, resulting in hyperglycemia, which may consequently lead to renal dysfunction [1,2]. The prevalence of diabetes-induced renal injury has been intensifying worldwide [3,4]. Approximately 30–50% of diabetes patients develop diabetic nephropathy, which has become a critical reason for the high morbidity and mortality rates [5]. In particular, diabetic nephropathy is a one of the leading factors in the expansion of end-stage renal disease (ESRD) in diabetes patients and is the causative factor for approximately 40% of new ESRD cases in both Western and Asian countries [6]. Diabetic nephropathy has been classically defined by the urinary excretion of microalbumin, and the stage is known as microalbuminuria [7]. Major pathological characteristics of diabetic nephropathy encompass glomerulosclerosis, interstitial fibrosis, and tubular atrophy [8]. Moreover, the accretion of advanced glycation end products (AGEs) in the blood may have an imperative role in the progression and development of diabetic nephropathy in patients via both oxidative stress and chronic inflammation [9,10].

The key pathological modifications of diabetic nephropathy consist of glomerular sclerosis, extracellular matrix (ECM) alterations, and tubulointerstitial fibrosis [11,12,13]. Furthermore, several findings revealed that inflammatory cytokines have diverse functions in the advancement of diabetic nephropathy [14,15]. Moreover, transforming growth factor-β (TGF-β), a well-known crucial regulator, contributes to the formation of kidney cell fibrosis in diabetic nephropathy [16]. The overexpression of TGF-β may stimulate the transition of epithelial–mesenchymal (EMT) and renal sclerosis, resulting in renal dysfunction [17,18] and indicating that the anti-TGF-β1 antibody exerts suppressive effects on fibrotic gene activation when administered after the onset of proteinuria in two distinct mouse models of glomerulonephritis. To date, many therapeutic drugs have been identified for the prevention of diabetic nephropathy. In spite of the widespread use of therapeutic approaches focused on hyperglycemia or diabetic nephropathy, abundant patients still continue to experience progressive or severe renal injury induced by diabetes. Therefore, it is essential to cultivate new medications for the protection of diabetic nephropathy.

The Hwangchil tree’s scientific name is *Dendropanax morbifera* (family Araliaceae), and this plant is an endemic tree species in the southern regions of Korea [19]. The most noted medicinal property of *D. morbifera* is restoring the immune system by continuously eliminating toxic substances from the body and revitalizing basic capabilities. This herb can be used to prepare a Korean tea, for easy consumption, and does not induce any side effect with prolonged use [19]. In Korea, different parts of *D.* *morbifera* in extract form have been used in traditional medicine for the treatment of headaches, skin diseases, and infectious diseases [20,21,22]. Methanolic extract obtained from the lower stem part of *D.* *morbifera* exhibits in vitro antiplasmodial activity [23], and essential oil extracted from flowers of *D.* *morbifera* claimed larvicidal activity against *Aedes aegypti* L. [24]. Since this extract has been known for its anti-diabetic properties [25], we inspected the scientific rationale and mechanism of action of water extract of *D.* *morbifera* (DP) against streptozotocin (STZ)-induced diabetic nephropathy.

Here, we investigated whether DP extract can attenuate diabetic nephropathy and renal fibrosis in STZ-induced diabetic rats. We hypothesized that DP extract attenuates diabetes-induced renal injury by rescuing hyperglycemia and pancreatic injury.

## 2. Materials and Methods 

### 2.1. Chemicals and Reagents

Streptozotocin had been obtained from Sigma Aldrich Biotechnology (St. Louis, MO, USA). Primary antibodies against pyruvate kinase muscle isozyme M2 (PKM2), kidney injury molecule-1 (KIM-1), selenium binding protein-1 (SBP1), neutrophil gelatinase-associated lipocalin (NGAL), collagen-1, fibronectin, alpha-smooth muscle antigen (α-SMA), Bcl-2-associated X protein (Bax), B-cell lymphoma 2 (Bcl-2), and β-actin had been obtained from Abcam (Cambridge, MA, USA). Primary antibodies against cleaved caspase-3, cleaved caspase-9, and β-actin had been obtained from Cell Signaling Biotechnology (Beverly, MA, USA). The horseradish peroxidase (HRP)-conjugated secondary antibody was obtained from Santa Cruz biotechnology.

### 2.2. Preparation of DP Extract from the Leaf and Stem of D. Morbifera

The leaf and stem of *D. morbifera* were collected in October 2017 at Gwangyang, Jeollanam-do, Korea. A voucher specimen was deposited at the School of Pharmacy, Sungkyunkwan University (specimen No.: SKKU-Ph-17-021). The dried leaf and stem (100 g) were extracted twice with water (1 L) at 90 °C for 5 h. The extract was concentrated under reduced pressure to prepare a water extract (DP extract) for further experiments. The DP extract was stored at 4 °C until further use. 

### 2.3. HPLC Analysis of the DP Extract

To characterize the major components of DP extract, quantitative analysis was directed using a reversed-phase (RP) C_18_ HPLC. HPLC analysis was implemented on a Knauer Smartline system consisting of a Manager 5000, Pump 1000 (× 2), a UV Detector 2500, and a Phenomenex Kinetex^®^ (Torrance, CA, USA) 5 μm C18 100Å column (150 × 4.6 mm). The eluent consisted of methanol (A) and 0.05% trifluoroacetic acid (TFA) in water (B). The gradient profile was 10% to 100% A in B for 45 min. The column oven temperature, flow rate, and UV absorption were set at 30 °C, 1 mL/min, and a 279 nm wavelength, respectively. Standard working solutions for four major compounds were prepared by serial dilution with a mixture solution of methanol and water (1:1) to give concentrations of 500, 200, 100, and 50 μg/mL, respectively. The contents of four major constituents detected as major peaks at 270 nm were determined using a regression equation for each compound. 

### 2.4. Assessment of Acute Toxicity of DP extract

To evaluate the toxicity profile of DP extract, healthy rats were distributed randomly into four groups. For 7 days, four different doses of aqueous extracts of DP (100, 500, 1000, and 3000 mg/kg/day) were administered orally. The animals were monitored continuously up to 7 days for any behavioral and clinical signs after DP extract exposure [26]. 

### 2.5. Experimental Design

Four-week-old Sprague–Dawley male rats were obtained from Charles River Laboratories (Orient, Seoul, Korea). The animals were retained in a room (specific pathogen-free (SPF)-conditioned) with a 12 h light/dark cycle. Ad libitum food (PMI, Brentwood, MO, USA) and tap water were provided to all animals. A single dose of STZ (60 mg/kg, freshly prepared in 0.1 mmol/L cold citrate buffer, pH 4.5) was injected intraperitoneally (i.p.). The animals that had fasting blood glucose levels greater than 300 mg/dL five days post-STZ treatment were diagnosed as diabetic and used for further experiments [27]. Five experimental groups were formed (*n* = 5): Group I: control rats received normal saline (NC), Group II: diabetic rats (STZ, 60 mg/kg, single i.p. injection), Group III: diabetic rats receiving DP (25 mg/kg), Group IV: normal rats only receiving DP (25 mg/kg, oral), and Group V: diabetic rats receiving metformin (50 mg/kg, oral). All procedures were carried out according to the guidelines of the Ethical Research Committee in Sungkyunkwan University (SKKU-2018-0065).

### 2.6. Assessment of Biochemical Parameters 

After the final drug treatment, all rats were fasted overnight and anesthetized with ketamine/xylazine (6.25 mg/1.25 mg/100 g weight by i.p.). Blood was collected from the inferior vena cava by opening the thoracic cavity and then centrifuged at 1500× *g* for 10 min. The serum was collected and stored at −80 °C until use. Serum was used to analyze asparate aminotransferase (AST), alanine aminotransferase (ALT), blood urea nitrogen (BUN), serum creatinine (sCr), triglyceride (TG), low-density lipoprotein (LDL), high-density lipoprotein (HDL), and advanced glycation end produce (AGE) using the VetScan analyzer (Abaxis Inc., CA, USA). 

### 2.7. Urinary Parameters Analysis

Metabolic cages were used to collect urine in glass bottles containing protease inhibitor. The supernatant was obtained from urine, centrifuged at 3000× *g* for 10 min, and stored at −20 °C for further use. Urinary pH was measured using Multistix 10SG reagent strips (Bayer, Elkhart, IN). Urinary glucose, creatinine, microalbumin, and protein levels were estimated by an automatic urine analyzer (Sysmex, Japan).

### 2.8. Histopathological Examination of Liver, Kidney, and Pancreas

Neutral formalin was used to fix all tissues (left kidney, liver, and pancreas) by keeping it in for 24 h at 25 °C and entrenched in paraffin and mounted on slides. The hematoxylin and eosin (H&E) staining was executed as previously described [28], with some modification. Histopathological images were obtained using a Zeiss Axiphot light microscope (Zeiss, Oberkochen, Germany) fitted with a Sony 3CCD camera (AVT Horn, Aalen, Germany). Histological findings were performed by double-blinded by Professor Whan Sup Cho and his colleague histologists (Dong-a University, Busan, Republic of Korea). Furthermore, Masson’s trichrome (MT) staining used to study the morphology of renal fibrosis as per the manufacturer’s protocol (Trichome, Gomori one-step, Aniline Blue Stain Kit, Newcomer Supply, Middleton, WI, USA). 

### 2.9. Immunohistochemical Analysis

Immunohistochemistry (IHC) was performed to evaluate TGF-β1, fibronectin, collagen-I, and α-SMA expression in renal tissue. After the deparaffinization of each slide, an antigen retrieval step was performed, and the slides were washed in Tris-buffered saline (TBS buffer) and incubated with primary antibodies including TGF-β1 (1:300), fibronectin (1:500), collagen I (1:500), and α-SMA (1:500) at 4 °C overnight. The sections were washed with TBS, followed by incubation with the HRP-conjugated secondary antibody (Santa Cruz, CA, USA) at room temperature for 30 min. The immunostaining was visualized using diaminobenzidine tetrahydrochloride (DAB), and the slides were counterstained with hematoxylin. Photomicrographs of kidney sections were obtained at 100× magnification with a confocal laser scanning microscope (K1-Fluo, Nanoscope System, Daejeon, Korea).

### 2.10. Analysis of Advanced Glycation end Products

The advanced glycation end products assay was executed accordingly as previously described [28]. This enzyme-linked immunosorbent assay (ELISA) was based on a competitive inhibition enzyme immunoassay technique. A precoated microplate with specific monoclonal antibody blocked with unknown (serum) and standard sample overnight. The unbound conjugate was washed, followed by incubation with HRP-conjugated secondary antibody for 1 h at room temperature. The color intensity created by adding a substrate solution for 2–20 min. The intensity developed was inversely proportional to the concentration of AGE in the sample. A microplate reader was used to determine absorbance at 450 nm.

### 2.11. Determination of 3-Indoxyl Sulfate (3-IS) 

3-indoxyl sulfate (a metabolite of tryptophan amino acid) is well-known uremic toxin that enhances renal injury via glomerular sclerosis and interstitial fibrosis. 3-indoxyl sulfate is also involved in reactive oxygen species (ROS) production in kidney cells, thus increasing oxidative stress. The amount of 3-IS in urine, serum, and kidney tissue was analyzed by high-performance liquid chromatography (HPLC). Organic solvent was used for the extraction of samples using a multistep process. After extraction, the samples were mixed with an internal standard (2-napthalenesulfonic acid) thoroughly and analyzed by HPLC (Gilson, LC-321 322 350) at 280 nm. The extraction recovery of samples was found to be 87%. 

### 2.12. Measurement of 4-Hydroxyproline Content

The partial hydrolysis of collagen provides a mixture of protein and peptides including 4-hydroxyproline (a marker indicating collagen levels). The measurement of 4-hydroxyproline has been used to identify certain diseases that involve the breakdown of collagen such as kidney injury. Hence, kidney tissue samples were used to determine the total amount of 4-hydroxyproline using a colorimetric assay kit (Cell Biolabs, San Diego, CA, USA).

### 2.13. Determination of Antioxidant Enzymes Activity and Oxidative Stress

Total glutathione and oxidized reduced glutathione (GSH) (GSSG) amounts were quantified in serum calorimetrically using the Glutathione Assay Kit (Cayman Chemicals Company, Ann Arbor, MI, USA). The ratio of the oxidized to reduced forms of glutathione (GSH/GSSG) was calculated after GSSG (oxidized glutathione) subtraction from the total glutathione to determine the amount of reduced GSH. The activity of superoxide dismutase (SOD) was quantified using a colorimetric SOD assay kit (Cayman Chemical, Ann Arbor, MI, USA). The peroxidation function of catalase (CAT) was assessed using a colorimetric assay kit (Cayman Chemical, Ann Arbor, MI, USA). Lipid peroxidation was determined by measuring the amount of thiobarbituric acid (TBA) reactive substances in serum using a commercial assay kit (TBARS assay kit, Cayman, USA). 8-Hydroxy-2’-deoxyguanosine (8-OHdG) is one of the predominant forms of free radical-induced oxidative DNA lesions, and it has therefore been widely used as a biomarker for oxidative stress [29]. Therefore, the urinary excretion of 8-OHdG is a reliable biomarker for detecting diabetes-induced oxidative stress. The amount of 8-OHdG in urine was measured using a competitive ELISA assay kit (Cell Biolabs, San Diego, CA, USA).

### 2.14. Determination of Inflammatory Cytokines in the Serum

Serum levels of pro-inflammatory mediators (TGF-β1, interleukin (IL)-1β, and IL-6) and an anti-inflammatory mediator (IL-10) were measured using specific assay kits according to the manufacture’s protocol (Abcam, Cambridge, MA, USA).

### 2.15. Terminal Deoxynucleotidyl Transferase dUTP Nick End Labeling (TUNEL) Assay

TUNEL assay was used to determine DNA fragmentation during apoptosis. The DeadEnd^TM^ colorimetric system (Promega, Madison, WI, USA) was used to detect apoptotic cells in the kidney tissue of diabetic rats.

### 2.16. Western Blot Analysis 

Total protein was extracted from frozen kidney tissue samples by lysis buffer. A BCA kit (Bio-Rad, Hercules, CA, USA) was used to quantify the protein concentration of homogenized tissue lysate according to the manufacturer’s instructions. Equal amounts of sample protein were subjected to 10% or 15% SDS-PAGE to separate different molecular weight proteins, and then they were transferred to Polyvinylidene difluoride membrane (PVDF) membranes by electroblotting. The PVDF membrane (Millipore, Billerica, MA, USA) was blocked with 5% skim milk at room temperature. Subsequently, the PVDF membranes were incubated with different primary antibodies overnight at 4 °C. Primary antibodies included: KIM-1 (1:500), NGAL (1:500), PKM2 (1:500), SBP1 (1:500), collagen-1 (1:1000), fibronectin (1:5000), vimentin (1:1000), α-SMA (1:1000), cleaved caspase-3 (1:1000), and cleaved caspase-9 (1:1000). The membranes were washed for 1 h with Tris buffer Saline solution (TBST) buffer and incubated with HRP-conjugated anti-mouse IgG (1:2000) or anti-rabbit IgG (1:1000) antibodies for 60 min at room temperature. The blots were developed using enhanced chemiluminescence (ECL)-plus kit (Amersham Biosciences, Buckinghamshire, UK).

### 2.17. Statistical Analysis

The results are expressed as the mean ± S.D. of six animals. Statistical significance for comparison between two groups was calculated using the two-tailed Student’s *t* test. To assess comparisons between multiple groups, statistical analysis was performed by one-way ANOVA followed by the Tukey’s HSD post hoc test for multiple comparisons using the Graph-Pad Prism 4 program (GraphPad Software, Inc, San Diego, CA, USA). The *p* value of 0.05 was considered to be statistically significant.

## 3. Results

### 3.1. Phytochemcials Characterization of DP Extract

**Figure 1:**
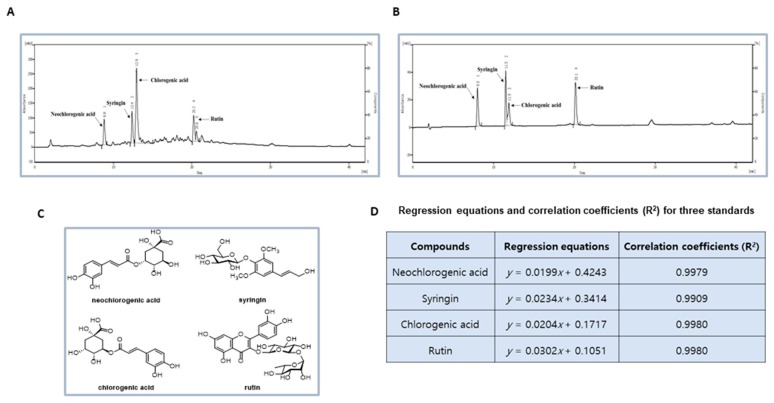
Preparation of *Dendropanax morbifera* water extract (DP) and experimental design for streptozotocin (STZ)-induced nephrotoxicity in rats. (**A**) High-performance liquid chromatography (HPLC)-UV chromatogram for DP extract. Major components in DP extract were identified and quantified by using HPLC analysis. HPLC analysis was carried out via gradient elution on a Phenomenex Kinetex C18 column (150 × 4.6 mm, 5 µm). The flow rate, column oven temperature, and UV wavelength for detection were set at 1 mL/min, 30 °C, and 254 nm, respectively. (**B**) Representative chromatogram on standard compounds (neochlorogenic acid, syringin, chlorogenic acid, and rutin). (**C**) The chemical structures of major compounds in DP extract and the regression equations and correlation coefficients for the four standard compounds. (**D**) The regression equation and correlation coefficient (R^2^) for three standards.

**Figure 2:**
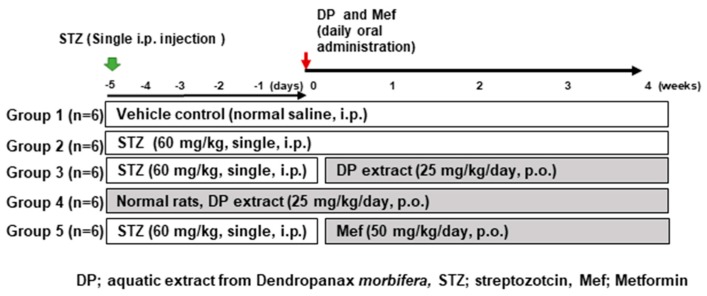
Experiment design. After one week of adaption, rats were divided randomly into five groups: (1) vehicle control (VC, *n* = 6); (2) STZ (60 mg/kg, single intraperitoneal (i.p.) injection); (3) STZ (60 mg/kg, single i.p. injection) + DP (25 mg/kg/day, oral gavage); (4) DP extract (25 mg/kg/day, oral gavage); and (5) STZ (60 mg/kg, single i.p. injection) + Mef (50 mg/kg/day, oral gavage). DP and Mef were administered daily for 4 weeks by oral gavage. STZ, streptozotocin; Mef, metformin.

As shown in the HPLC chromatograph (Figure 1A), there are four major peaks observed from DP extract, including neochlorogenic acid, syringin, chlorogenic acid, and rutin by comparison with standard materials (Figure 1B,C). The contents of four major components in the DP extract were determined as 6.56, 7.11, 39.42, and 7.01 μg/mg, respectively by using regression equations (Figure 1D). Figure 2 represents the experimental design of the DP extract in the Sprague–Dawley male rats.

### 3.2. Acute Oral Toxicity Study of DP Extract

The DP extract was tested for the acute toxicity for 7 days by various doses. After monitoring the animals for 7 days routinely, there were no signs of any abnormal behavior or toxicity or mortality associated with DP extract administration noticed. Table 1 describes in detail the acute oral toxicity data.

### 3.3. Effect of DP Extract on Body Weight, Glucose Concentration, Organ Weight, and Histology in STZ-Induced Diabetic Rats

**Figure 3:**
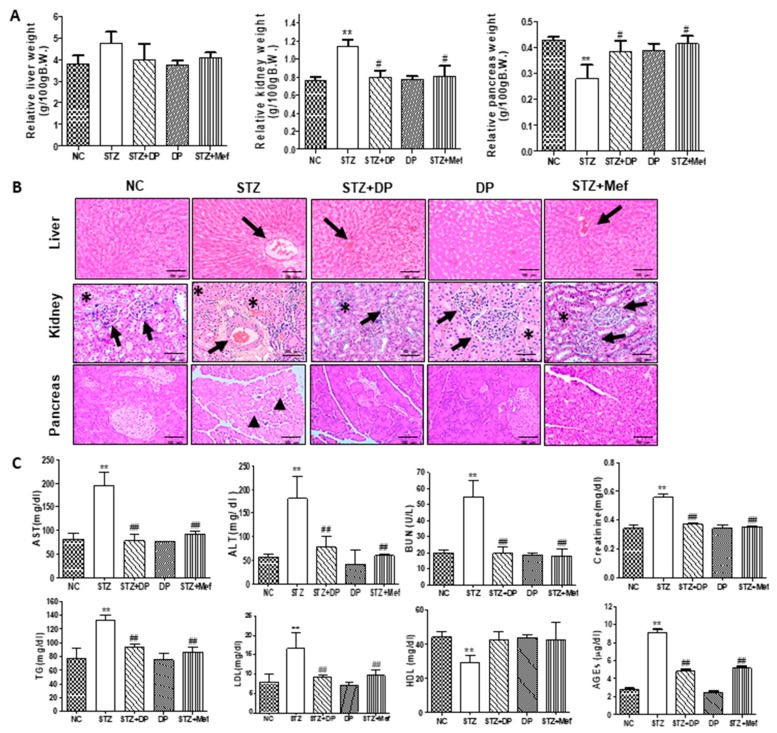
Effect of DP extract on relative organ weight, histopathological changes, and biochemical parameters in STZ-induced diabetic rats. (**A**) Relative organ weight changes in STZ-induced diabetic rats. Values are expressed as the mean ± S.D. for six rats. Statistical analysis was performed by one-way ANOVA followed by Tukey’s HSD post hoc test for multiple comparisons. ** *p* < 0.01 compared to vehicle control; ^#^ *p* < 0.05 compared to STZ-treated group. (**B**) Representative histology of hematoxylin and eosin (H&E) stained liver, kidney, and pancreatic sections from experimental groups. STZ-induced rats showed enlarged cortex with glomerular sclerosis (arrowheads) and expansion (asterisk), and they also showed dilatation. The cortex from DP extract-treated rats displayed a normal histological structure of tubules and collecting ducts. The islet cells are seen interspersed between the acinar cells. The islets appeared more lightly stained than the surrounding acinar cells. STZ-induced diabetic rats revealed pathological changes in the acinar cells, which were observed as swollen and small vacuoles (black arrowheads). Islet β-cells are almost entirely lost in STZ-induced diabetic rats. However, the administration of DP extract showed distortion of the general architecture. An atrophic change of the acinar cells was less severe, and the border between exocrine and endocrine portions became more distinct. Images are representative of three animals per experimental group (magnification 100×). (**C**) Effect of DP extract on biochemical parameters. Values are expressed as the mean ± S.D. for six rats. Statistical analysis was performed by one-way ANOVA followed by Tukey’s HSD post hoc test for multiple comparisons. ** *p* < 0.01 compared to vehicle control; ^##^ *p* < 0.01 compared to the STZ-treated group.

There were no significant differences in clinical signs between treatment groups during the experimental periods. However, a significant reduction in the body weight gains was found in STZ-treated rats (Table 2). The relative kidney weight was significantly increased in STZ-treated rats, whereas the administration of DP extract or metformin recovered kidney weight (Figure 3A). The relative pancreas weight was significantly reduced in the STZ-treated rats compared with that of the control group. However, the administration of DP extract or metformin markedly prevented changes in relative pancreas weights (Figure 3A). No significant differences in the relative liver weights were observed between any treatment groups (Figure 3A). As shown in Table 3, fasting blood glucose levels gradually increased in STZ-induced diabetic rats. In contrast, the administration of DP extract or metformin significantly reduced blood glucose levels back to normal values in STZ-induced diabetic rats. 

Figure 3B demonstrates the representative histopathological changes in liver, kidney, and pancreatic tissues. There were no obvious differences in histological characteristics between the control and DP extract groups. However, tubulointerstitial injuries and glomerular damage were apparently observed in the kidney of STZ-induced diabetic rats. As expected, these renal injuries observed in STZ-induced diabetic rats were protected by the administration of DP extract for 28 days (Figure 3B). Metformin also markedly protected against tubular injuries in diabetic rats (Figure 3B). In particular, histopathological alterations in the pancreas were clearly observed for STZ-induced diabetic rats. DP extract or metformin administration resulted in the marked recovery of diabetes-induced pancreatic damages (Figure 3B). In addition, enlarged hepatocytes containing Mallory bodies due to the degeneration of hepatic cells observed in STZ-induced diabetic rats were clearly recovered after the administration of DP extract (Figure 3B). Diabetic rats showed an enlarged cortex with glomerular sclerosis (arrowheads) and expansion (asterisk), and they also showed dilatation. The cortex from DP extract-treated rats demonstrated a normal histological structure of tubules and collecting ducts. The islet cells are seen interspersed between the acinar cells. The islets appeared more lightly stained than the surrounding acinar cells. Swollen and small vacuoles (black arrowheads) observed in acinar cells directed the pathological changes in diabetic rats. Islet β-cells are almost entirely lost in STZ-induced diabetic rats. However, the administration of DP extract showed distortion of the general architecture. The atrophic change of the acinar cells was less severe, and the border between exocrine and endocrine portions became more distinct (Figure 3B). These results suggest that the hepatic-renoprotective effects of DP extract against damage observed in STZ-induced diabetic rats were closely related to the regulation of blood glucose levels via the regeneration of pancreatic function.

### 3.4. Effect of DP Extract on Biochemical Parameters in Diabetic Rats

We measured serum AST, ALT, BUN, sCr, triglyceride (TG), LDL, HDL, and AGE levels (Figure 3C). There were significant elevations in AST, ALT, LDL, and TG in STZ-induced diabetic rats, but the administration of DP extract significantly reduced these biochemical parameters, and the efficacy of DP extract was similar to that observed as a result of the administration of metformin. It is worth mentioning that the administration of DP extract reduced TG and LDL levels in diabetic rats to levels similar to those of the control rats, indicating that DP extract also improves lipid homeostasis. In STZ-induced diabetic rats, AGE levels were increased significantly compared to those of the control. However, the administration of DP extract or metformin to these rats significantly reduced the AGE levels (Figure 3C). These results strongly suggest that DP extract ameliorates the accumulation of AGE via the regulation of blood glucose levels in STZ-induced diabetic rats. Furthermore, STZ-induced diabetic rats also showed a significant increase in BUN and sCr levels, which are nephrotoxicity biomarkers that were recovered by the administration of DP extract or metformin (Figure 3C). 

### 3.5. Effect of DP Extract on Urinary Excretion of Kidney Injury Biomarkers in Diabetic Rats

**Figure 4:**
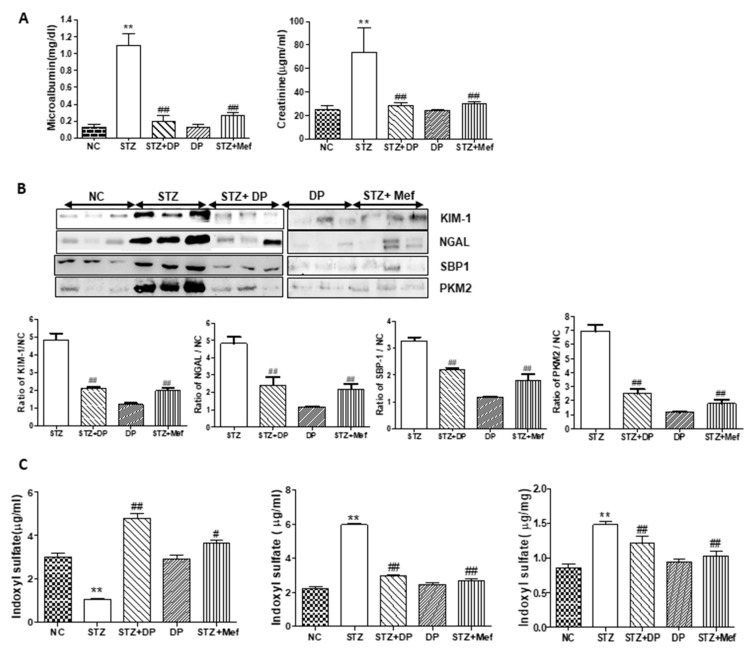
Effect of DP extract on changes in kidney injury biomarkers in the urine of STZ-induced diabetic rats. (**A**) Changes in urinary microalbumin and creatinine. Values are expressed as the mean ± S.D. for 6 rats. Statistical analysis was performed by one-way ANOVA followed by the Tukey’s HSD post hoc test for multiple comparisons. ** *p* < 0.01 compared to vehicle control; ^##^ *p* < 0.01 compared to STZ-treated group. (**B**) Changes in the urinary excretion of kidney injury biomarkers in STZ-induced diabetic rats by the administration of DP and metformin. Representative Western blots of kidney injury molecule-1 (KIM-1), neutrophil gelatinase-associated lipocalin (NGAL), selenium binding protein-1 (SBP1), and pyruvate kinase muscle isozyme M2 (PKM2) levels in the urine of rats. Statistical analysis was performed by one-way ANOVA followed by Tukey’s honestly significant difference test (HSD) post-hoc test for multiple comparisons. ** *p* < 0.01 compared to vehicle control; ^#^ *p* < 0.05, and ^##^ *p* < 0.01 compared to the STZ-treated group. (**C**) Changes in the levels of 3-indoxyl sulfate (3-IS) in STZ-induced diabetic rats after treatment with DP extract and metformin. Concentration of 3-IS in the urine, serum, and kidney tissues was measured by HPLC analysis. Values are expressed as the mean ± S.D. for 6 rats. Statistical analysis was performed by one-way ANOVA followed by Tukey’s HSD post hoc test for multiple comparisons. ** *p* < 0.01 compared to vehicle control; ^#^ *p* < 0.05 and ^##^ *p* < 0.01 compared to the STZ-treated group.

In STZ-induced diabetic rats, urinary microalbumin and Cr levels were significantly increased compared to those of the control. However, the administration of DP extract or metformin in these rats drastically reduced microalbumin and Cr levels (Figure 4A). These results clearly indicate that DP extract protects against diabetes-induced nephrotoxicity. In order to validate the diabetic nephropathy, we also measured the urinary expression of KIM-1, NGAL, SBP1, and PKM2, which are supposed new biomarkers for detecting renal injury. As shown in Figure 4B, urinary excretion of the respective proteins levels was significantly increased in STZ-induced diabetic rats compared with those of the control, while the administration of DP extract or metformin markedly reduced KIM-1, SBP1, PKM2, and NGAL levels in the urine and almost restored them to those of the control rats (Figure 4B). We also measured 3-IS levels as markers of AKI in the urine, serum, and kidney. Generally, 3-IS is a metabolite of dietary tryptophan, and our previous study demonstrated that high concentrations of 3-IS in the serum and kidney tissues were closely related with kidney injury [30]. As shown in Figure 4C, 3-IS levels were significantly increased in the serum and kidney tissue, while the urinary excretion level was reduced. The administration of DP extract or metformin drastically recovered 3-IS concentrations in the serum, kidney, and urine to levels similar to those observed in control rats. The results also provided clear evidence for the protective effects of DP extract against diabetic nephropathy.

### 3.6. Effect of DP Extract on ROS Production, Antioxidant Enzyme Activity, and Oxidative Stress in the Kidney of Diabetic Rats

**Figure 5:**
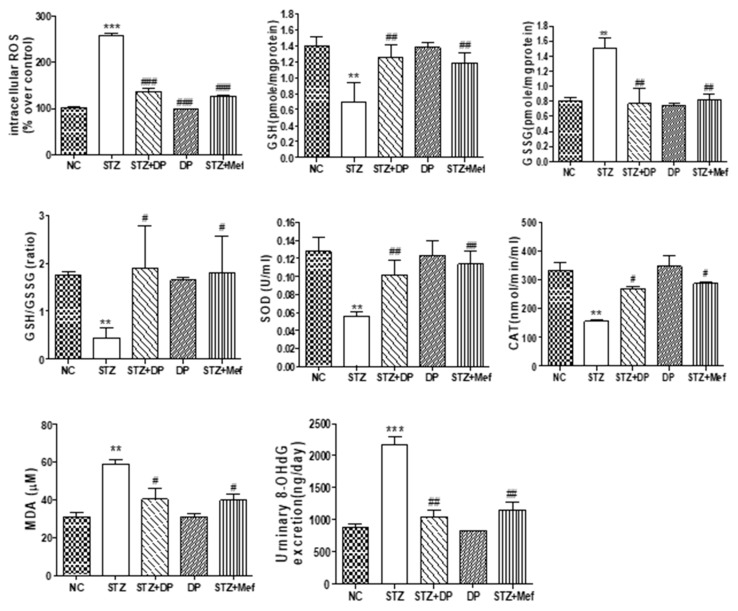
Effect of DP extract on antioxidant enzyme activity and oxidative stress in the kidney of STZ-induced diabetic rats. Changes in oxidative biomarkers (reduced glutathione (GSH), glutathione and oxidized GSH (GSSG), GSH/GSSG, superoxide dismutase (SOD), and catalase) were measured in the kidney. Intracellular reactive oxygen species (ROS) and oxidative DNA damage marker 8-Hydroxy-2’-deoxyguanosine (8-OHDG) were measured in the kidney. Data are expressed as the means ± S.D. of duplicate experiments (6 animals/group). Statistical analysis was performed by one-way ANOVA followed by Tukey’s HSD post hoc test for multiple comparisons. *** *p* < 0.001 and ** *p* < 0.01 compared to control group; ^#^ *p* < 0.05 and ^##^ *p* < 0.01, ^###^ *p* < 0.001 compared to STZ-treated group.

We determined the effects of DP extract on diabetes-induced oxidative stress. Actually, diabetes is defined as excessive ROS production surpassing the existing anti-oxidative defense mechanisms, and it plays a critical role in the development and progression of diabetic nephropathy [31,32]. STZ-induced diabetic rats showed drastically increased blood glucose levels. Interestingly, hyperglycemia increased ROS production in the kidney of diabetic rats compared with that of normal rats (Figure 5). However, the administration of DP extract and metformin significantly inhibited ROS production nearly up to normal levels. These results indicated that the nephroprotective effect of DP extract is related to the inhibition of ROS production by participating in regulation antioxidant enzymes activity. In order to evaluate the possible mechanisms underlying the effects of DP extract on diabetes-induced oxidative stress, antioxidant enzyme activity was measured in the kidneys of diabetic rats. As shown in Figure 5, we clearly observed a significant reduction in GSH and the induction of GSSG levels in the kidney of STZ-induced diabetic rats. However, the administration of DP extract for 4 weeks showed a significant improvement in GSH levels, supporting the notion that the protection of kidney injury was closely associated with defense against oxidative stress in diabetic rats (Figure 5).

Antioxidant enzymes are important for protecting the cell from oxidative damage by ROS. The activities of SOD and CAT in renal tissue are shown in Figure 5. SOD and CAT activities were markedly reduced in STZ-induced diabetic rats compared with those in the control group. However, DP extract treatment significantly increased SOD and CAT activities compared to those in the diabetic group (Figure 5). Malondialdehyde (MDA) is a by-product of lipid peroxidation, which causes oxidative stress in diabetic rats. The level of MDA was significantly elevated in diabetic rats compared to that of normal rats. Treatment with DP extract for 4 weeks significantly decreased MDA levels in diabetic rats to levels similar to those observed for rats treated with metformin. 8-OHdG is the product of oxidative DNA damage, which is a biomarker of oxidative stress. In STZ-induced diabetic rats, the urinary excretion of 8-OHdG markedly increased, indicating the high generation of ROS and damage to DNA in the kidney. The administration of DP extract for 4 weeks significantly decreased the urinary excretion of 8-OHDG in diabetic rats (Figure 5).

### 3.7. Effect of DP Extract on Production of Pro-Inflammatory Cytokines in Diabetic Rats

**Figure 6:**
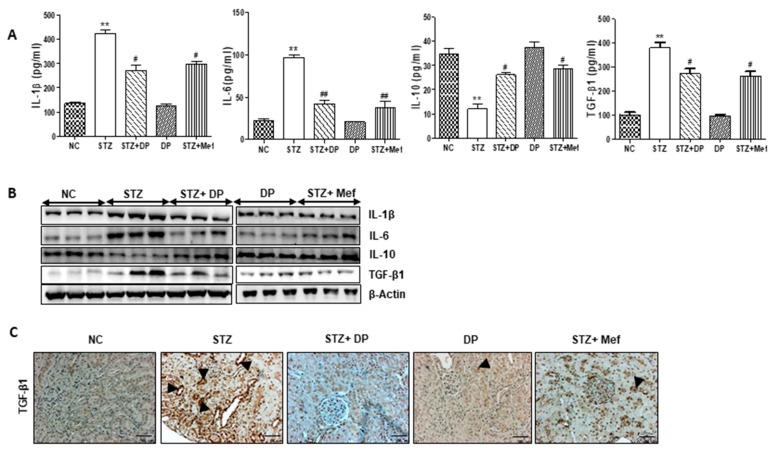
Effect of DP extract on inflammatory cytokines levels in the serum of STZ-induced diabetic rats. (**A**) Serum levels of cytokines were measured using assay kits. Data are expressed as the means ± S.D. of duplicate experiments (6 animals/group). Statistical analysis was performed by one-way ANOVA followed by Tukey’s HSD post hoc test for multiple comparisons. ** *p* < 0.01 compared to control group; ^#^ *p* < 0.05 and ^##^ *p* < 0.01 compared to STZ-treated group. (**B**) Expression of interleukin (IL)-1β, IL-6, IL-10, and TGF-β1 was measured in the kidney of STZ-induced diabetic rats by Western blot analysis. β-Actin expression was used as the loading control. The Western blot results are representative of three separate experiments. (**C**) Representative immunohistochemical staining of transforming growth factor-β (TGF-β1) in the kidney of STZ-induced diabetic rats. Black arrows represent TGF-β1 expression. Original magnification: 100×.

To evaluate the possible roles of DP extract in the regulation of pro-inflammatory cytokines in diabetic rats, we measured the secretion of TGF-β1, IL-1β, IL-6, and the anti-inflammatory cytokine, IL-10, in serum. As shown in Figure 6A, TGF-β1, IL-1β, and IL-6 levels were significantly increased in the serum of diabetic rats compared to those of normal control. However, the secretion of IL-10 was markedly reduced. DP extract administration significantly reduced pro-inflammatory cytokine production in diabetic rats, while IL-10 levels were elevated. As shown in Figure 6B, the expression of these pro-inflammatory cytokines was confirmed in the kidney tissues. Moreover, the expression of TGF-β1, an exclusive fibrotic cytokine, in kidney tissue was drastically increased in diabetic rats (Figure 6C). However, the administration of DP extract and metformin markedly inhibited TGF-β1 expression in these rats. Therefore, these data indicate that DP extract significantly suppresses the secretion of inflammatory cytokines and increases the secretion of an anti-inflammatory cytokine in diabetic rats.

### 3.8. Effect of DP Extract on Apoptosis in the Kidney of Diabetic Rats

**Figure 7:**
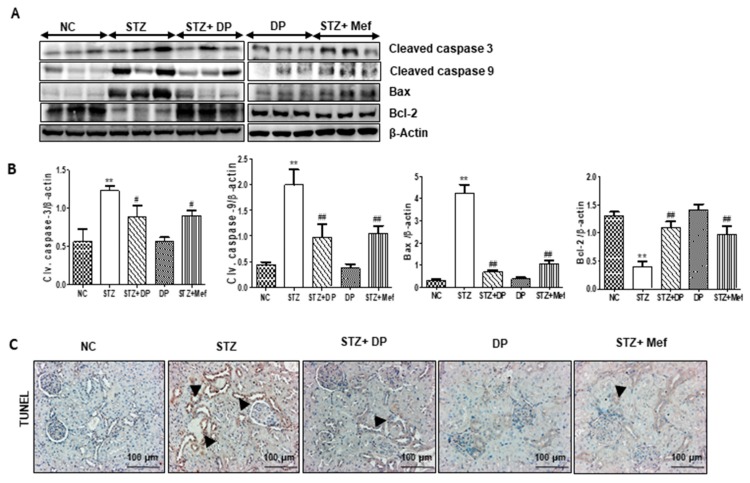
Effect of DP extract on apoptosis in the kidney tissues of STZ-induced diabetic rats. (**A**) Expression levels of cleaved caspase 3, cleaved caspase 9, Bcl-2-associated X protein (Bax), B-cell lymphoma 2 (Bcl-2) were measured by Western blot analysis, using an experimental model of STZ-induced diabetic rats. β-Actin expression was used as the loading control. The Western blot results are representative of three separate experiments. (**B**) Representative graphs indicate the fold changes of Western blot data. Values are the mean ± S.D. of triplicate experiments. Statistical analyses were performed using one-way ANOVA followed by Tukey’s HSD post hoc test for multiple comparisons. ** *p* < 0.01 compared to the control group; ^#^ *p* < 0.05 and ^##^ *p* < 0.01 compared to the STZ-treated group. (**C**) Representative immunohistochemical staining of transferase dUTP nick end labeling (TUNEL) in the kidney of STZ-induced diabetic rats. Black arrows represent TUNEL-positive cells. Original magnification: 200×, scale bar: 50 μm.

To determine the protective effect of DP extract on diabetic nephropathy, we measured the expression of Bax, cleaved-caspase 3, and cleaved-caspase 9, which are upregulated in the kidney of diabetic rats, promoting apoptotic cell death. However, treatment with DP extract and metformin markedly reduced the expression of these apoptosis-related proteins. In addition, treatment with DP extract significantly enhanced the expression of Bcl-2 (Figure 7A,B). The TUNEL assay confirmed these results. As shown in Figure 7C, TUNEL positive nuclear staining was drastically increased in the kidney of STZ-induced diabetic rats, which demonstrates the kidney damage caused by the induction of the apoptotic pathway. However, the administration of DP extract and metformin drastically reduced the number of TUNEL-positive nuclei in STZ-induced diabetic rats.

### 3.9. Effect of DP Extract on the Composition of ECM in the Kidney of Diabetic Rats

**Figure 8:**
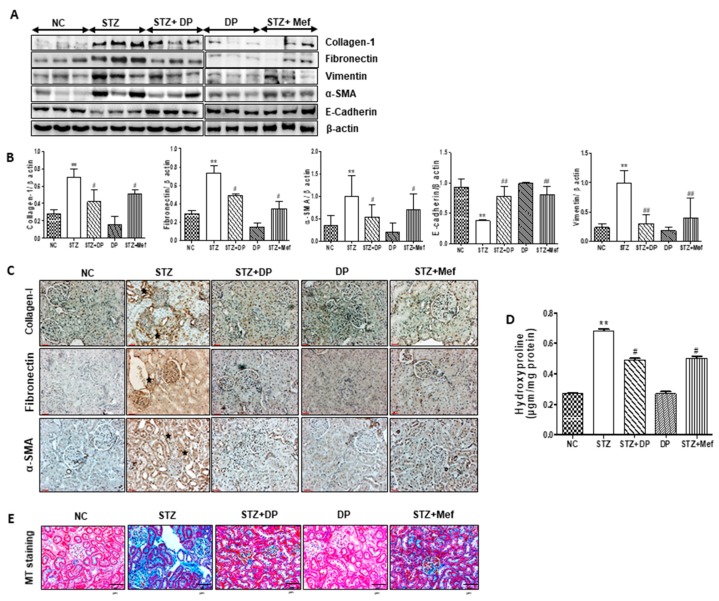
Effect of DP extract on renal fibrous biomarkers in STZ-induced diabetic rats. (**A**) Expression levels of E-cadherin, collagen-1, alpha-smooth muscle antigen (α-SMA), α-tubulin, TGF-β, vimentin, and fibronectin were measured by Western blot analysis, using an experimental model of STZ-induced diabetic rats. β-Actin expression was used as the loading control. The Western blot results are representative of three separate experiments. (**B**) Representative graphs indicate the fold changes of Western blot data. Values are the mean ± S.D. of triplicate experiments. Statistical analyses were performed using one-way ANOVA followed by Tukey’s HSD post hoc test for multiple comparisons. ** *p* < 0.01 compared to the control group; ^#^ *p* < 0.05 and ^##^ *p* < 0.01 compared to the STZ-treated group. (**C**) Representative immunohistochemical staining of collagen-1, fibronectin, and α-SMA in the kidney of STZ-induced diabetic rats. Stars represent collagen-1, fibronectin, and α-SMA-positive expression. Original magnification: 200×, scale bar: 100 μm. (**D**) 4-Hydroxyproline content in the serum of STZ-induced diabetic rats. Data are expressed as the means ± S.D. of duplicate experiments (6 animals/group). Statistical analysis was performed by one-way ANOVA followed by Tukey’s HSD post hoc test for multiple comparisons. ** *p* < 0.01 compared to control group; ^#^ *p* < 0.05 compared to STZ-treated group. (**E**) Representative Masson’s trichrome-stained kidney sections. Blue color represents collagen accumulation. Original magnification: 200×, scale bar: 100 μm. Original magnification: 100×, scale bar: 100 μm.

In order to explore the protective effect of DP extract on diabetes-induced renal fibrous, ECM composition and 4-hydroxyproline levels were measured in the kidney of diabetic rats. As shown in Figure 8A,B, the expression of collagen-1, fibronectin, vimentin, and α-SMA was significantly increased in diabetic rats. Furthermore, the expression of E-cadherin was downregulated. In addition, these results were confirmed by IHC (Figure 8C). As expected, collagen-1, α-SMA, and fibronectin expression was highly increased in the glomeruli and renal interstitial area of diabetic rats, while their expression was decreased in rats administered DP extract. The administration of DP extract significantly reduced the expression of these proteins, which confirmed the amelioration of the ECM in the kidney (Figure 8B). To further support these findings, 4-hydroxyproline levels were also investigated in the kidney tissue of diabetic rats. As shown in Figure 8D, the level of 4-hydroxyproline was markedly increased in diabetic rats, while the administration of DP extract reduced the level of 4-hydroxyproline in kidney tissue. These observations were demonstrated by a significant increase in the MT-stained positive area in diabetic renal sections when compared to that of the normal controls. We found that collagen fibers had significantly accumulated in the STZ-induced diabetic rats (Figure 8E). Consistent with the changes observed in the glomerulus, treatment with DP extract markedly reduced the collagen deposition that was observed in the renal interstitial in diabetic rats. 

## 4. Discussion

The incidence of diabetes is gradually increasing, and complications of diabetes are considered to be a serious public health concern. Moreover, therapeutic options for diabetic nephropathy are limited, which is a growing problem worldwide. As a complementary and alternative medicine, traditional herbal medicine and its main components have been extensively investigated to develop safe and effective drugs for the treatment and prevention of diabetic nephropathy [33,34]. 

The standardization of DP extract was achieved by HPLC analysis for four major compounds. The major components of DP extract including neochlorogenic acid, syringin, chlorogenic acid, and rutin were profiled and quantified successfully. In particular, neochlorogenic acid, syringin, chlorogenic acid, and rutin isolated from different sources have been shown to have numerous biological and pharmacological activities. Among these compounds, chlorogenic acid is defined as the major component responsible for the biological activity against renal toxicity, nephropathy, and renal fibrosis [27,35,36]. In addition, rutin also has been investigated for the treatment of nephropathy [37,38]. *D. morbifera* has been reported to have beneficial effects on diabetes [25]. Recent studies have suggested that *D. morbifera* solvent extract inhibits high glucose-induced mesangial cell proliferation and ECM accumulation [25,39]. However, the underlying preventive mechanism of *D. morbifera* extract in diabetic nephropathy remains unclear. The present study explored the molecular mechanism of DP extract on protection against nephropathy in STZ-induced diabetic rats. Our experimental study elucidated that the sCr and BUN levels were significantly increased in STZ-induced diabetic rats, which indicates that these rats exhibited severe kidney injury. Furthermore, STZ-treated rats exhibited significant elevation in blood glucose levels, relative kidney weight, urinary excretion of renal injury biomarkers or microalbumin, inflammatory cytokines, and renal fibrosis, which are indicators of renal dysfunctions. However, the administration of DP extract for 28 days drastically ameliorated the effects to these renal injury biomarkers, and thus DP extract protected against diabetes-induced renal injury.

To confirm whether DP extract attenuates diabetic nephropathy, we measured sensitive renal injury biomarkers because the major target site of diabetes is tubular dysfunction, which precedes glomerular injury. Thus, the determination of urinary excretion of sensitive biomarkers may be helpful in understanding the protective mechanism of DP extract against diabetes-induced renal injury [40]. The over/under expression of urinary biomarkers guided toward different phases of kidney pathology in chronic diabetes. In the early detection of renal injury, SBP1 is a worthwhile biomarker, while NGAL reflects the inflammatory status of kidney cells during nephrotoxicity. The overexpression of KIM-1 also outlines the tubular injury in kidney [41,42]. In the present study, the administration of DP extract significantly reduced the urinary excretion of NGAL, KIM-1, SBP1, and PKM2 in STZ-induced diabetic rats. Therefore, DP extract attenuated the early stage of diabetes-induced renal injury. However, DP extract alone did not cause any renal dysfunction, which indicates that DP extract has no adverse effects on kidney function in rats.

There is strong evidence that chronic inflammation and oxidative stress are associated with the progression of kidney dysfunction and hyperglycemia [43,44]. Under hyperglycemic conditions, ROS production clogs kidney cells [45]. Thus, the excessive production of ROS depletes the antioxidant defenses, which results in the oxidation of DNA, lipids, and proteins in the kidney. In addition, hyperglycemia attenuates anti-oxidative mechanisms through the glycation of scavenging enzymes, such as SOD and catalase [46]. In addition, ROS also generate due to the interaction of glucose with protein results in AGEs, which blocked the receptors and inactivated the enzymes [47]. Since AGEs were found to be involved in the progression of many irreversible complications such as diabetes, the amount of AGEs was also considered an important marker to estimate during nephrotoxicity [48]. To investigate whether DP extract could protect against hyperglycemia-induced nephropathy, we measured the blood glucose levels in STZ-treated rats. Our data revealed that the administration of DP extract or metformin significantly reduced blood glucose levels in STZ-induced diabetic rats. Additionally, oxidative stress via changes in antioxidant balance was recovered to normal levels in the kidneys of STZ-induced diabetic rats by the administration of DP extract. These results clearly show that oxidative damages, such as the formation of MDA and 8-OhdG, were drastically reduced by the administration of DP extract. In recent years, 8-OhdG, the oxidative form of DNA, also appears as the most frequent measurable marker among the oxidative stress markers to indicate DNA damage [29]. Considerable evidence suggests that hyperglycemia-induced ROS formation is closely associated with kidney dysfunction. In particular, excessive ROS can induce apoptosis in renal tubule cells [49]. Our study provided evidence that the apoptosis was more obvious in the kidneys of STZ-induced diabetic rats. Thus, high blood glucose levels gradually increased ROS production, which led to renal dysfunction, similar to that observed in previous studies [26,28]. As diabetic nephropathy involves multiple signaling pathways, therapeutic strategies should focus on pharmacological agents with multidimensional effects. 

Nowadays, antioxidant therapy may be important for optimizing protection against renal fibrosis in diabetes patients [50,51]. Inflammatory cytokines have also been linked to the initiation of renal fibrosis in diabetes patients [14]. Similar to the previous studies [20], we demonstrated that DP extract and metformin significantly reduced IL-1β and IL-6 and improved IL-10 expression in the serum of diabetic rats, indicating that DP extract possesses anti-inflammatory activity. Furthermore, chronic inflammation drives immune cell activation and the subsequent activation of intrinsic renal cells, with the subsequent production and release of profibrotic cytokines and growth factors leading to the renal fibrosis [52]. Therefore, renal fibrosis is the final outcome of progressive diabetic nephropathy, and it results in a significant disruption of kidney structure and function. Generally, TGF-β1 is upregulated in response to inflammatory stimuli, causing renal fibrosis and decomposition of the EMT in the renal tubules [53]. Furthermore, TGF-β1 increases collagen synthesis contributing to ECM accumulation, leading ultimately to glomerulosclerosis in diabetes patients [54]. Similarly, our present study suggests that DP extract can inhibit TGF-β1 expression in the renal cortex of diabetic rats. Concomitantly, collagen-1 and fibronectin levels were significantly reduced in the kidney of diabetic rats after the administration of DP extract for 28 days. Therefore, we suggest that DP extract downregulates TGF-β1 expression and reduces the accumulation of ECM in diabetic rats. These results were confirmed again by the observation that DP extract also effectively diminished the 4-hydroxyproline level in the serum of diabetic rats, suggesting the protective mechanism of DP extract against renal fibrosis in diabetes-induced rats. 

## 5. Conclusions

We demonstrated that the oral administration of DP extract reduces the blood glucose concentration in STZ-induced diabetic rats. Finally, DP ameliorates renal injury thorough the inhibition of ROS generation and mitochondrial apoptosis in the kidneys of diabetic rats. These findings suggest the potential biological activity of DP on the progression of diabetes nephropathy. Further studies are required to understand the specific signaling pathway through which the components of DP exert its protective effects against the diabetic nephropathy.

## Figures and Tables

**Table 1:** Acute oral toxicity data.

Dose	Day 1	Day 2	Day 3	Day 4	Day 5	Day 6	Day 7
Group 1 (100 mg/kg/day)	120.67 ± 0.58	120 ± 2.65	118.33 ± 5.13	121.67 ± 1.154	119 ± 1	114.67 ± 3.05	121 ± 2
Group 2 (500 mg/kg/day)	119.67 ± 1.163	115.33 ± 1.02	121.33 ± 3.05	120 ± 1.13	119 ± 2	116.67 ± 1.71	121 ± 3.11
Group 3 (1000 mg/kg/day)	121.67 ± 1.51	122.33 ± 2.30	120 ± 1	120.67 ± 0.57	117 ± 2.54	120.33 ± 2.21	121 ± 1.08
Group 4 (3000 mg/kg/day)	120.33 ± 2.23	118 ± 2	120.33 ± 1.154	119.33 ± 1.01	119 ± 1	119.33 ± 1.50	120.68 ± 2.51

**Table 2:** Effect of *Dendropanax* (DP) on body weight in diabetic-induced nephrotoxicity rats after 28 days of treatment.

Group	Week 0	Week 1	Week 2	Week 3	Week 4	Relative Growth Rate (%)
NC	211.65 ± 5.38	219.13 ± 8.23	231.67 ± 4.01	251.17 ± 4.57	267.4 ± 6.41	26.3 (i/c)
STZ	211.67 ± 6.39	211.43 ± 8.01	202.93 ± 1.50 ^a^	193.63 ± 2.23 ^a^	184.33 ± 5.64 ^a^	−12.9 (d/c)
DP	213 ± 2.08	221.47 ± 1.56	231.87 ± 2.04 ^b^	250.6 ± 2.07 ^b^	269.66 ± 1.53 ^b^	26.4 (i/c)
STZ + DP	212.63 ± 5.83	216.25 ± 1.43	225.98 ± 1.84 ^b^	245.83 ± 0.28 ^b^	255.19 ± 5.15 ^b^	20.01 (i/c)
STZ + Mef	214.34 ± 4.04	223.3 ± 5.81	229.53 ± 7.51 ^b^	240.63 ± 1.46 ^b^	252.03 ± 2.61 ^b^	17.5 (i/c)

Values are presented as means ± SD. *n* = 5; One way analysis of variance (ANOVA) was used for the comparison of means. Differences between means were significant at *p* < 0.05 using Bonferroni’s multiple comparison test. ^a^ *p* < 0.05 in comparison with normal control group; ^b^ *p* < 0.05 in comparison with the STZ (diabetic control) group. i/c-Increase;d/c-decrease. NC: normal saline.

**Table 3:** Effect of *Dendropanax* (DP) on blood glucose level in diabetic-induced nephrotoxicity rats after 28 days of treatment.

Group	Week 0	Week 1	Week 2	Week 3	Week 4	Change (%)
NC	131 ± 8.01	132.33 ± 1.52	129.33 ± 1.15	130.33 ± 1.527	131.66 ± 2.08	0.50 (i/c)
STZ	423.33 ± 40.05 ^a^	515 ± 61.55 ^a^	563.33 ± 32.15 ^a^	593 ± 11.27 ^a^	599.33 ± 0.58 ^a^	41.57 (i/c)
DP	134 ± 4.36 ^a^	127.66 ± 3.21 ^a,b^	128.66 ± 1.15 ^b^	129.66 ± 0.57 ^b^	129 ± 4.58 ^b^	−3.73(d/c)
STZ + DP	432 ± 44.50 ^b^	318.33 ± 96.02 ^a,b^	233.66 ± 104.04 ^b^	150 ± 26.46 ^b^	131.67 ± 9.07 ^b^	−69.52 (d/c)
STZ + Mef	422 ± 32.36 ^a^	347.33 ± 40.77 ^a,b^	258.67 ± 4.51 ^a,b^	171.67 ± 6.03 ^a,b^	139 ± 7.21 ^b^	−67.06 (d/c)

Values are presented as means ± SD. *n* = 5; one way analysis of variance (ANOVA) was used for the comparison of means. Differences between means were significant at *p* < 0.05 using the Bonferroni’s multiple comparison test. ^a^ *p* < 0.05 in comparison with the normal control group; ^b^ *p* < 0.05 in comparison with STZ (diabetic control) group. i/c, Increase; d/c, Decrease.

## Abbreviations

AGEs: advanced glycation end products; ALT, alanine aminotransferase; AST, aspartate aminotransferase; AKT, acute kidney injury; BUN, blood urea nitrogen; DAB, diaminobenzidine tetrahydrochloride; ECM, extracellular matrix; EMT, epithelial–mesenchymal transition; GSH, reduced glutathione; HRP, horseradish peroxidase; HPLC, high-performance liquid chromatography; 3-IS, 3-indoxyl sulfate; KIM-1, kidney injury molecule-1; MDA, malondialdehyde; NGAL, neutrophil gelatinase-associated lipocalin; ROS, reactive oxygen species; PI3K, phosphatidylinositol-3-kinases; PKM2, pyruvate kinase muscle isozyme M2; SBP1, selenium binding protein-1; sCr, serum creatinine; α-SMA, alpha-smooth muscle antigen; SOD, superoxide dismutase; SPF, specific pathogen free; STZ, streptozotocin; TGF-β, transforming growth factor-β.
